# Exercising in Times of Lockdown: An Analysis of the Impact of COVID-19 on Levels and Patterns of Exercise among Adults in Belgium

**DOI:** 10.3390/ijerph17114144

**Published:** 2020-06-10

**Authors:** Bram Constandt, Erik Thibaut, Veerle De Bosscher, Jeroen Scheerder, Margot Ricour, Annick Willem

**Affiliations:** 1 Department of Movement and Sports Sciences, Ghent University, Watersportlaan 2, 9000 Ghent, Belgium; 2 Department of Movement Sciences, KU Leuven, Tervuursevest 101, 3001 Leuven, Belgium; erik.thibaut@kuleuven.be (E.T.); jeroen.scheerder@kuleuven.be (J.S.); 3 Department of Sport Policy and Management, Vrije Universiteit Brussel, Pleinlaan 2, 1050 Brussels, Belgium; veerle.de.bosscher@vub.be (V.D.B.); margot.ricour@vub.be (M.R.)

**Keywords:** COVID-19, health promotion, pandemic, physical exercise, sport participation

## Abstract

Countries all over the world implemented lockdowns to counteract COVID-19. These lockdowns heavily limited people’s exercise possibilities. At the same time, experts advocated to remain physically active to prevent future health problems. Based on an online survey, this study examines adults’ exercise levels and patterns during the COVID-19 lockdown in Belgium. Ordinal logistic regression analyses of 13,515 valid and population-weighted responses indicate a general increase in exercise frequencies, as well as in sedentary behavior. Except for people aged 55+, previously low active adults self-reported to exercise more during the lockdown. Among the people who were already high active before COVID-19, those above 55 years old, those with low education, those used to exercise with friends or in a sport club, and those who were not using online tools to exercise, self-reported to exercise less during the lockdown. Having less time, sitting more, and missing the familiar way and competitive element of exercising were the main reasons for a self-reported exercise reduction. Given the health risks associated with physical inactivity, results imply that governments should consider how those who were not reached can be encouraged to exercise during a lockdown. After all, additional COVID-19 lockdowns might be implemented in the future.

## 1. Introduction

Aiming to slow the spread of COVID-19, many countries across the globe turned to restrictive policy measures, by which the freedom of movement of their citizens was heavily limited. However, at the same time, experts argued in favor of continued exercise during this crisis to avoid health problems, such as increasing obesity, depression, infections, and cardiovascular diseases, as much as possible [1]. To deal with this health-related duality caused by COVID-19, different countries implemented different sorts of lockdown scenarios, with varying degrees of freedom of movement. While countries such as China, India, New Zealand, Italy, and France enforced very strict measures in terms of how (far) people could physically move outside their homes, banning any “unnecessary” public outdoor activity, Belgium opted for a so-called “lockdown light” [2,3].

During this lockdown light, of which the first exercise-related measures started on 13 March 2020, and which were only gradually being attenuated as of 4 May 2020, schools were closed, and working from home became the new standard whenever possible. Furthermore, citizens were allowed and even encouraged by the government to exercise, but with considerable restrictions. More precisely, citizens were encouraged to exercise in their homes (e.g., by doing yoga, dancing, and bodyweight training) [4], and to exercise outdoors, limited to walking, running, cycling, and unmotorized activities on wheels (e.g., skateboarding, roller skating, and kick scootering), alone, with members of the same household, or with one friend. There was no distance limit, but taking a car or motorcycle to go exercising was not allowed. Stops, taking a rest, and paying visits to others during exercise were also forbidden. In addition, with all fitness and health centers and sport clubs closed, and sport competitions postponed or even cancelled, organized sport faced an extensive crisis, with multifold harmful economic and social consequences [5,6].

Despite these clear and drastic changes, there are currently no scientific insights into how people changed their exercise levels and patterns because of the specific lockdown situation in their country. Therefore, this paper’s aim is to examine whether adults remained or started exercising during the lockdown, whilst paying attention to the demographic and exercise-related characteristics of the high active and low active people, as well as to the underlying reasons for exercising less or more. Three research questions are put forward: what is the influence of the COVID-19 lockdown on adults’ exercise behaviors in Flanders? (*RQ 1*); what are the demographic and exercise-related characteristics of those who reported exercising less, as much, or more? (*RQ 2*); and what are the reasons that help to explain why people are exercising less, as much, or more? (*RQ 3*). Gaining knowledge about these factors is of the utmost importance, because being physically active by means of exercise might considerably influence one’s ability to cope with the COVID-19 crisis by reducing stress and anxiety, while increasing immunity, well-being, and quality of sleep and life [4]. Furthermore, as it is unlikely that we will return to the “old normal” in the near future, it is important to scientifically inform (future) policies about the impact of a lockdown on exercise levels and patterns.

## 2. Materials and Methods

### 2.1. Study Design and Procedure

A cross-sectional study design was implemented. Approximately two weeks after the start of the first lockdown measures on 13 March 2020, an online survey of exercise behaviors of Flemish (i.e., the largest, Dutch-speaking, northern part of Belgium) citizens was developed. After ethical clearance of an independent ethics committee at KU Leuven (for adult participants only), the survey, conducted via the web-based Qualtrics software (Qualtrics, Provo, UT, USA), was randomly dispersed to as many people as possible from 30 March until 5 April 2020. Subject recruitment was done with the help of Flanders’ most read newspaper *Het Laatste Nieuws* (i.e., by means of news articles—both online and on paper—mentioning this study and the accompanying online survey), and with support from public and private sport organizations’ social media and e-mailing channels. Respondents had to confirm their informed consent prior to completing the online survey.

### 2.2. Measures

The online survey consisted of several questions measuring (a) whether adults were exercising less, as much, or more during the lockdown (i.e., a closed-ended question—ordinal variable—with three response categories); (b) the characteristics of their exercise levels and patterns before and after the lockdown (i.e., closed-ended questions with yes/no/not applicable response options, such as “are/were you exercising alone, with your partner/family, with friends, with online support, in a sport club?”); (c) the experienced obstacles (i.e., closed-ended questions with yes/no/not applicable response options, such as “fearful to get COVID-19”, “closed sport infrastructure”, “no sport club activities”, “no interest (anymore)”, “no good environment”, “less/little time”, “being ill”, and “cancelled sport events”); (d) aspects of their former activities they missed most (i.e., closed-ended questions with yes/no/not applicable response options, such as “do you miss exercising with friends?”, “do you miss your familiar way of exercising?”, and “do you miss the competition element of exercising?”); and (e) sedentary behavior (i.e., a closed ended-question—ordinal variable—with three response categories: sitting less, as much, more). Exercise characteristics and obstacles were selected based on existing research (e.g., the Flemish sport participation survey) [7,8,9]. Demographics, such as gender (i.e., male/female) age (i.e., categories 18–34, 35–54, 55–74 years old), highest educational level achieved (i.e., student higher education, high school, higher education), and the inhouse presence of children (i.e., yes/no) were also measured.

### 2.3. Data Analyses

To retain a representative population sample, the collected data of the adults were checked for geographical dispersion and, after post-stratification for age (i.e., older than 75 years), weighted in terms of sex, age, highest educational level, and household situation (i.e., inhouse presence of children). Recent population and survey statistics—also based on the age category 18–75—formed the basis for this selection [8]. Finally, 13,515 valid responses were withheld for further analyses, by means of descriptive and ordinal logistic regression analyses. These latter analyses were computed separately for the high active (i.e., those who exercised regularly/at least once a week before COVID-19) and low active (i.e., those who exercised non-regularly/less than once a week, including non-participation before COVID-19) groups in SPSS 26 (IBM, Armonk, NY, USA). This categorization is in line with previous international research on exercising, the Flemish sport participation survey, and the guidelines of the World Health Organization concerning regular physical activity (i.e., at least once a week) [8]. Demographic variables (i.e., gender, age category, and inhouse presence of children) and exercise-related variables (i.e., exercising alone, with partner/family, with friends, with online support, in a sport club) thereby functioned as factors, while self-reported exercising less, as much, or more during the lockdown operated as the three ordinal categories of the dependent variable.

## 3. Results

### 3.1. Descriptive Statistics

A total of 15,737 people fully completed the survey. An overview of the frequencies of the demographic variables of the final, cleaned sample (*n* = 13,515) can be found in Table 1. High active people were strongly overrepresented in the sample (*n* = 11,763, equaling 87% of the total sample). Therefore, all further analyses were conducted separately for the two subsamples of high active (*n* = 11,763) and low active (*n* = 1752) people. Initial frequencies analyses of exercise patterns were executed for both subsamples. Table 1 demonstrates in more detail that both subsamples are distinct in terms of their demographic constitution.

#### 3.1.1. Subsample High Active People (Pre COVID-19)

Among the high active people, 36% reported higher exercising levels than before the lockdown, 41% reported exercising as much as before, and 23% reported exercising less. Approximately half of the high active people (54%) considered that they had more time than before to exercise (6% less time, 36% as much time). Concerning sedentary behavior, 46% indicated to sit more, 39% to sit as much, and 15% to sit less than before. The major exercise obstacles experienced by this subsample related to closed sport infrastructure (50%), the non-presence of sport club activities (38%), cancelled sport events (32%), and the absence of friends to exercise with (30%) (see Table 1).

#### 3.1.2. Subsample Low Active People (Pre COVID-19)

Among those that were classified as low active people before the COVID-19 lockdown, 58% were exercising more, 5% as much, and 7% less during the lockdown than in the period before the lockdown. The remaining 30% did not really exercise at all during the lockdown. A total of 61% of the low active people found more time to exercise than prior to the lockdown (8%: less time; 24%: as much time). For sedentary behavior, 40% signaled that they sit more, 36% sit as much, and 24% sit less than before the lockdown. The main obstacles obstructing these people’s engagement in exercise were being fearful for contamination with COVID-19 (29%), closed sport infrastructure (27%), the absence of friends to exercise with (21%), and a lack of interest (20%) (see Table 1).

### 3.2. Ordinal Logistic Regression Analyses

Three multivariate ordinal logistic regression analyses were executed to analyze the demographics of exercising less, as much, or more during the lockdown in the low active subsample, and to analyze demographics and exercise-related characteristics, as well as potential reasons for exercising more during the lockdown in the high active subsample.

#### 3.2.1. Subsample High Active People (Pre COVID-19)

The model of a first multivariate ordinal logistic regression looking into the potential influence of demographic and exercise-related factors has a significant and good fit (*χ*^2^ (11) = 318.672, *p* = 0.000). Approximately three percent of the variance in exercising less, as much, or more during the lockdown on behalf of the pre COVID-19 active people can be explained by the variance in the included factors (Nagelkerke *R*^2^ = 0.032). Table 2 provides an overview of the regression coefficients for each factor. As shown, people in the age categories 18–34 and 35–54 are more likely to fall in a higher exercising category during the lockdown, compared to people in the age category 55–74. Furthermore, people whose highest educational level is high school have a smaller chance of residing in a higher exercising category, in comparison with people who successfully completed higher education.

People who were not exercising with their family or partner, and those who were not exercising with the support of online tools were less likely to end up in a higher exercise category during the lockdown, when compared, respectively, with those who did sometimes exercise with their family or partner, and those who were already exercising with the support of online tools (e.g., running or cycling apps such as Strava, Zwift, and Runkeeper). On the other hand, people who were exercising with friends and those who were exercising in a sport club had a smaller chance of falling into a higher exercise category during the lockdown, compared, respectively, with those who were not exercising with friends, and those who were not engaged in a sport club.

A number of significant reasons for changes in exercise levels during compared to before the lockdown was found (see Table 3). A total of 27% of the variance in moving less, as much, or more during the lockdown is explained by this model (Nagelkerke *R*^2^ = 0.271), while the model has a good fit (*χ*^2^ (14) = 1643.355, *p* = 0.000). Firstly, having less or as much time to exercise during the lockdown (compared to before) lowers one’s chance of falling within a higher exercise category. Second, people who reported less and as much sedentary behavior compared to before the lockdown were more likely to end up in a higher exercise category. Finally, people who were missing their familiar way of exercising and people who were missing the competitive aspect of exercising were less likely to exercise more during the lockdown.

#### 3.2.2. Subsample Low Active People (Pre COVID-19)

Model fitting information of this multivariate ordinal logistic regression indicates a significant and good fit of our tested model (*χ*^2^ (8) = 158.251, *p* = 0.000). Moreover, 18.5% of the variance in exercising less, as much, or more during the lockdown on behalf of the pre COVID-19 low active people can be explained by the variance in the independent variables (Nagelkerke *R*^2^ = 0.185). As demonstrated in Table 4, people in the age categories 18–34 and 35–54 were more likely to fall in a higher exercise category during the lockdown, compared to people in the age category 55–74. Furthermore, low active people who reported less and as much sedentary behavior were more likely to end up in a higher exercise category.

## 4. Discussion

The global spread of COVID-19 has a strong impact on exercise levels and patterns all over the world. In Belgium, people were encouraged to exercise non-intensively during the lockdown to stimulate their mental and physical health [4]. Whereas other countries (e.g., China) encouraged people to exercise in their houses during the lockdown, both inhouse exercising and outdoor exercising in public spaces were promoted in Belgium [2]. Nonetheless, the Belgian lockdown heavily reduced people’s range of opportunities to exercise. This simultaneous combination of exercise promotion and restriction could influence both people who were high active and low active before the lockdown. Further, the results of this study might nurture our understanding of how to avoid a serious temporary and potentially permanent population reduction in physical activity in times in which protecting the population’s health by installing a lockdown is unavoidable.

While approximately one third (36%) of the high active people was exercising more during the lockdown (41% as much), almost two-thirds of the low active people (58%) indicated higher exercise levels. (*RQ 1*). Additionally, certain demographic and exercise-related determinants of changed exercise levels were observed (*RQ 2*). In the low active sample, being older and sitting more contributed to exercising less. Moreover, part of the explanation for exercising less on behalf of the high active subsample was found in having less time, sitting more, missing one’s familiar way of exercising, and missing the competitive element of exercise (*RQ 3*).

Behavioral theory provides insights into why the respondents in our study might have exercised more or less. A closer examination of reported obstacles highlights the influence of temporarily restricted habits (i.e., exercising with friends and exercising in a sport club), showcasing that new social norms might lead to (forced) behavioral changes. Yet, self-determination theory suggests these changes risk being little sustainable, as they are rooted in extrinsic motivations (i.e., lockdown regulations and restrictions) rather than in intrinsic motivations [10]. Further, the significant results on exercise determinants support the influence of self-efficacy, social support, and social norms on self-perceived exercise obstacles and motivations [11]. Moreover, they also shed light on the different exercise functions or benefits (e.g., increasing health and facilitating and stimulating social contacts) [12]. COVID-19 puts the health benefits of exercise at the forefront of attention, while heavily limiting the social benefits. However, the inability to exercise with friends did not account for an exercise reduction. Hence, the health benefits of exercise seem important enough to keep high active people active, and to stimulate low active people to be more active. This result can be linked with social cognitive theory, as the environmental influence (i.e., threatened health), rather than the social influence, accounts for changed exercise levels and patterns during a lockdown [10].

Although the global spread of COVID-19 presents a unique situation, results on the negative effect of older age and low educational level are in line with existing research on exercise determinants [8,13,14]. The abovementioned results also support the belief that the interests and goals of people who exercise in a non-organized form differ significantly from those who exercise in a sport club [15]. This further implies that organized sport (e.g., in sport clubs) is required to stimulate a part of the population to exercise that would otherwise be difficult to motivate. As demonstrated by previous research, sport clubs contribute strongly to obtaining higher exercise levels in the population [16]. To illustrate its relevance post COVID-19, organized sport should emphasize the distinctive aspects of their operation (e.g., organizing competition and offering exercise habits) that contributed to people exercising less during the lockdown [16,17]. Sport clubs could thereby highlight their health promotion potential [18].

While the majority of all respondents indicated exercising as much or more during the lockdown, nearly half of them also reported sitting more. This result is not without risk, as a positive balance between both behaviors is required from a health perspective [19]. Regardless of the extent of physical activity, too much sedentary behavior is unhealthy, and can lead to not only physical but also mental health issues [20]. This result should be an important concern for policy makers, as the lockdown itself was leading to mental stress (e.g., by a distorted work–life balance and by strongly reduced social contacts) [21]. Moreover, the abovementioned results also suggest that exercise promotion during the COVID-19 lockdown did not successfully reach 55+ year old adults. Tailor-made exercise campaigns for this age group might be required. Conversely, highly active people might exercise too intensively during the lockdown, negatively impacting their immune system and making them more vulnerable towards COVID-19 [22]. Further, exercise support by coaches, physiotherapists, and others was strongly limited during the lockdown. However, virtual and online exercise support was possible. Moreover, highly active people who were used to exercise with online support before the lockdown were more likely to exercise more during the lockdown. This finding reinforces the idea that health and sport apps might help to stimulate exercise [23].

Although this study reached a large sample of respondents (*n* = 13,515), certain limitations are present. For example, the subsample of highly active people was clearly overrepresented, which could partly be related to our definition of highly active people. Future research could adopt a broader perspective, including other, less structured, and non-planned forms of physical activity beyond exercise during a lockdown. Nevertheless, more low active people were reached than in previous exercise research in Belgium. In addition, this issue of overrepresentation was taken care of by analyzing both subsamples separately. The unbalanced presence of highly active respondents is caused by a self-selection bias, which is inherent to the online survey method. Moreover, our approach to use closed-ended questions could bias respondents into giving a certain response, although response categories like “other” or “not applicable” were included to avoid forcing respondents into certain response options. Furthermore, since our study was executed only two weeks after the start of the lockdown, future scholarship is encouraged to look into potential long-term effects by means of longitudinal study designs. After all, people experiencing a (personal) crisis might adopt sustainable healthy habits (such as exercising) [24]. Behavioral and institutional theory could support such an analysis, helping to better understand (the sustainability of) changes in exercise [10,25].

## 5. Conclusions

Promoting inhouse exercise and exercise in public spaces during a lockdown might prevent an exercise reduction. However, not everyone increased their exercise levels during the COVID-19 lockdown in Belgium. Those exercise habits that were temporarily forbidden help explain why some people exercised less. Behavioral theory suggests that changes in exercise patterns and levels due to the lockdown might lack sustainability, due to not being intrinsically motivated nor socially influenced. Furthermore, this study implies that exercise support (e.g., online tools) and organized sport are recommended to exercise safely and to fully exploit all functions or benefits of exercise. Moreover, given the health risks associated with physical inactivity, the results also suggest that governments should consider how 55+ year old citizens, people with low education, and sport club participants can be encouraged to exercise during a lockdown. After all, additional COVID-19 lockdowns (or other countermeasures) might be implemented in the near future.

## Figures and Tables

**Table 1:** Participant demographics (*n* = 13,515).

Variable	Total Sample (*n* = 13,515)		Subsample High Active People (*n* = 11,763)		Subsample Low Active People (*n* = 1752)	
**Gender**	*n*	%	*n*	%	*n*	%
Male	6685	49.5	6189	52.6	496	28.3
Female	6831	50.5	5574	47.4	1256	71.7
**Age category**	*n*	%	*n*	%	*n*	%
18–34	3666	27.1	3000	25.5	666	38.0
35–54	5110	37.8	4569	38.8	541	30.9
55–74	4739	35.1	4194	35.7	545	31.1
**Highest education**	*n*	%	*n*	%	*n*	%
Student higher education	762	5.6	617	5.2	144	8.2
High school degree	8121	60.1	6970	59.3	1152	65.7
Higher education degree (ref.)	4633	34.3	4176	35.5	457	26.1
**Inhouse children**	*n*	%	*n*	%	*n*	%
Yes	5299	39.2	4552	38.7	747	42.6
No	8216	60.8	7211	61.3	1005	57.4
**Geographical region**	*n*	%	*n*	%	*n*	%
Antwerp	4387	32.5	3849	32.7	538	30.7
Limburg	1361	10.1	1206	10.3	155	8.8
East Flanders	3091	22.9	2613	22.2	479	27.3
Flemish Brabant	2270	16.8	2021	17.2	249	14.2
West Flanders	1997	14.8	1731	14.7	266	15.2
Brussels Capital	147	1.1	121	1.0	26	1.5
Other	263	1.9	222	1.9	41	2.3
**Reported exercise obstacles**	*n*	%	*n*	%	*n*	%
None	4181	33.6	3389	30.2	792	65.4
Fear for COVID-19	1362	13.1	1087	11.5	275	29.0
Closed infrastructure	4952	47.7	4720	49.6	232	27.0
No friends	3059	29.5	2878	24.5	181	21.4
No interest (any more)	449	4.3	259	2.8	190	20
No good environment	1855	18.1	1711	18.2	144	17.1
No sport club activities	3794	36.6	3659	38.4	135	16.2
No/little time	496	4.8	375	4.0	121	13.7
Illness	280	2.7	221	2.4	59	7.1
Cancelled sport event	3103	29.9	3026	31.6	76	9.5
Other (not specified)	693	6.8	583	6.2	110	12.9

**Table 2:** Output ordinal logistic regression analysis of the influence of demographic and exercise-related determinants on exercising less, as much, or more (subsample high active people, *n* = 11,763).

Variable	Estimate	Std. Err.	Wald	Sig. (*p*)	95% Confidence Interval
**Gender**					
Male	−0.044	0.036	1.469	0.225	[−0.115; 0.027]
Female (ref.)					
**Age category**					
18–34	0.428	0.051	71.668	0.000	[0.329; 0.527]
35–54	0.206	0.047	19.641	0.000	[0.115; 0.298]
55–74 (ref.)					
**Highest education**					
Student higher education	0.101	0.090	1.262	0.261	[−0.075; 0.277]
High school degree	−0.079	0.038	4.202	0.040	[−0.154; −0.003]
Higher education degree (ref.)					
**Inhouse children**					
No	−0.066	0.041	2.513	0.113	[−0.147; 0.016]
Yes (ref.)					
**Exercising alone**					
No	−0.037	0.040	0.841	0.359	[−0.115; 0.042]
Yes (ref.)					
**Exercising with partner/family**					
No	−0.196	0.038	26.127	0.000	[−0.271; −0.121]
Yes (ref.)					
**Exercising with friends**					
No	0.119	0.036	10.586	0.001	[0.047; 0.190]
Yes (ref.)					
**Exercising with online support**					
No	−0.281	0.058	23.380	0.000	[−0.394; −0.167]
Yes (ref.)					
**Exercise in a sport club**					
No	0.427	0.036	137.911	0.000	[0.356; 0.499]
Yes (ref.)					

Note: Dependent variable: exercise during lockdown, including three categories: i.e., exercising less, exercising as much, and exercising more.

**Table 3:** Output ordinal logistic regression analysis of potential reasons for exercising less, as much, or more (subsample high active people, *n* = 11,763).

Variable	Estimate	Std. Err.	Wald	Sig. (*p*)	95% Confidence Interval
**Gender**					
Male	0.122	0.052	5.530	0.019	[0.020; 0.225]
Female (ref.)					
**Age category**					
18–34	0.429	0.075	32.543	0.000	[0.281; 0.576]
35–54	0.246	0.068	12.906	0.000	[0.112; 0.380]
55–74 (ref.)					
**Highest education**					
Student higher education	0.000	0.120	0.000	0.999	[−0.235; 0.235]
High school degree	−0.263	0.055	23.230	0.000	[−0.371; −0.156]
Higher education degree (ref.)					
**Inhouse children**					
No	−0.090	0.059	2.295	0.130	[−0.206; 0.026]
Yes (ref.)					
**Time to exercise**					
Less	−2.505	0.126	395.408	0.000	[−2.752; −2.258]
As much	−1.024	0.056	336.494	0.000	[−1.134; −0.915]
More (ref.)					
**Sedentary behavior**					
Less	1.598	0.086	345.349	0.000	[1.429; 1.766]
As much	1.126	0.057	389.668	0.000	[1.014; 1.237]
More (ref.)					
**Missing familiar way of exercising**					
No	0.747	0.054	188.653	0.000	[0.640; 0.853]
Yes (ref.)					
**Missing competition**					
No	0.238	0.063	14.071	0.000	[0.114; 0.362]
Yes (ref.)					
**Missing exercising with others**					
No	−0.065	0.065	0.997	0.318	[−0.192; 0.063]
Yes (ref.)					
**Missing drinking with others after exercise**					
No	−0.055	0.056	0.970	0.325	[−0.165; 0.055]
Yes (ref.)					

Note. Dependent variable: exercise during lockdown, including three categories: i.e., exercising less, exercising as much, exercising more.

**Table 4:** Output ordinal logistic regression analysis of demographic determinants on exercising less, as much, or more (subsample low active people, *n* = 1752).

Variable	Estimate	Std. Err.	Wald	Sig. (*p*)	95% Confidence Interval
**Gender**					
Male	0.196	0.185	1.120	0.290	[−0.167; 0.560]
Female (ref.)					
**Age category**					
18–34	1.230	0.220	31.300	0.000	[0.799; 1.661]
35–54	0.896	0.230	15.119	0.000	[0.444; 1.348]
55–74 (ref.)					
**Highest education**					
Student higher education	0.328	0.385	0.723	0.395	[−0.428; 1.083]
High school degree	−0.341	0.188	3.227	0.070	[−0.710; 0.028]
Higher education degree (ref.)					
**Inhouse children**					
No	0.309	0.195	2.526	0.112	[−0.72; 0.691]
Yes (ref.)					
**Sedentary behavior**					
Less	2.174	0.248	76.981	0.000	[1.688; 2.659]
As much	1.512	0.202	56.219	0.000	[1.117; 1.908]
More (ref.)					

Note. Dependent variable: exercise during lockdown, including three categories: i.e., exercising less, exercising as much, exercising more.
